# Incremental Cost of Conducting Population-Based Prevalence Surveys for a Neglected Tropical Disease: The Example of Trachoma in 8 National Programs

**DOI:** 10.1371/journal.pntd.0000979

**Published:** 2011-03-08

**Authors:** Chaoqun Chen, Elizabeth A. Cromwell, Jonathan D. King, Aryc Mosher, Emma M. Harding-Esch, Jeremiah M. Ngondi, Paul M. Emerson

**Affiliations:** 1 Georgia Institute of Technology, Atlanta, Georgia, United States of America; 2 The Carter Center, Atlanta, Georgia, United States of America; 3 The London School of Hygiene and Tropical Medicine, London, United Kingdom; 4 Department of Public Health and Primary Care, Institute of Public Health, University of Cambridge, Cambridge, United Kingdom; University of Oklahoma Health Sciences Center, United States of America

## Abstract

**Background:**

Trachoma prevalence surveys provide the evidence base for district and community-wide implementation of the SAFE strategy, and are used to evaluate the impact of trachoma control interventions. An economic analysis was performed to estimate the cost of trachoma prevalence surveys conducted between 2006 and 2010 from 8 national trachoma control programs in Africa.

**Methodology and Findings:**

Data were collected retrospectively from reports for 165 districts surveyed for trachoma prevalence using a cluster random sampling methodology in Ethiopia, Ghana, Mali, Niger, Nigeria, Sudan, Southern Sudan and The Gambia.

The median cost per district survey was $4,784 (inter-quartile range [IQR] = $3,508–$6,650) while the median cost per cluster was $311 (IQR = $119–$393). Analysis by cost categories (personnel, transportation, supplies and other) and cost activity (training, field work, supervision and data entry) revealed that the main cost drivers were personnel and transportation during field work.

**Conclusion:**

Population-based cluster random surveys are used to provide the evidence base to set objectives and determine when elimination targets have been reached for several neglected tropical diseases, including trachoma. The cost of conducting epidemiologically rigorous prevalence surveys should not be a barrier to program implementation or evaluation.

## Author Summary

The costs of conducting population-based prevalence surveys for neglected tropical diseases such as trachoma are often cited as a reason that program managers do not conduct baseline or impact assessments when guidelines suggest they are warranted. The authors conducted a review of actual costs incurred during the implementation of 165 district level surveys in 8 national trachoma control programs to identify the median and mean costs per district and per cluster. In addition, the costs of the principal activities that are the most expensive were measured. The data show that field work is the most expensive activity for a prevalence survey, with personnel (*per diems*, allowances and accommodation) and transport costs driving the total cost of the survey. These findings can be used by program managers to budget for population-based prevalence surveys that are recommended for baseline and evaluation surveys, and periodic uptake surveys for neglected tropical diseases such as trachoma.

## Introduction

Trachoma is an eye disease, caused by infection with ocular *Chlamydia trachomatis,* which causes blindness. However, trachoma can be treated and prevented through the SAFE strategy, endorsed by the World Health Organization (WHO): Surgery for trichiasis; Antibiotic therapy through mass distribution; Facial cleanliness promotion through health education; and Environmental improvement with sanitation. Trachoma is endemic in 57 countries worldwide, with the burden of disease concentrated in sub-Saharan Africa and the Middle East[1]. The WHO estimates that over 80 million people currently have active trachoma and another 8 million suffer from trichiasis, with a potential productivity loss of $2.9 billion annually at the global scale[2]. The World Health Assembly has set 2020 as the target date for the elimination of blinding trachoma worldwide[3].

Where trachoma is suspected to be a public health problem, the WHO recommends that the prevalence of the clinical signs of the disease are estimated using a cluster random survey methodology at the district level[4]. There are two other less common methods used to assess the burden of trachoma disease: trachoma rapid assessments (TRA); and acceptance sampling trachoma rapid assessment (ASTRA)[5], [6]. As demonstrated in the literature[7], the population-based probability sampling (PBPS) method is the most epidemiologically robust method available to generalize the prevalence of clinical signs to the domain of interest.

In brief, the PBPS method employs a multi-stage cluster random survey design to randomly select clusters, and households within the clusters. Once households are selected, all members of the household are examined for clinical signs of trachoma disease using the WHO Simplified Grading System[8]. Survey team members are trained to conduct trachoma grading and household selection before participating in survey field work. Most survey teams consist of pairs of trachoma examiners and recorders, with one or two pairs needed to survey a cluster. Upon completion, double entry of survey data and analysis are performed by temporary staff or non-governmental organizations and Ministry of Health personnel.

Trachoma prevalence surveys provide an estimate of the burden of disease at the level of interest, usually the district. These data serve as the evidence base for determining how the SAFE strategy should be employed. For example, where the prevalence of the clinical grade TF (trachomatous inflammation, follicular) exceeds 10% in children aged 1–9 years, the WHO recommends district-wide mass treatment with antibiotics and facial cleanliness and environmental improvements—the “AFE” of SAFE. Prevalence survey data are also used to calculate annual intervention targets and ultimate intervention goals (UIGs), such as the number of people who require trichiasis surgery. These targets are used to plan annual activity budgets, forecast the need for donated pharmaceuticals and other supplies, and monitor progress towards the elimination of blinding trachoma.

Although survey implementation may vary by location, there are currently no data on the cost of trachoma prevalence surveys in the peer-reviewed literature. There are examples in the literature where different survey methods were compared to determine the most cost-effective method to estimate immunization coverage[9], [10]. While comparisons such as these can be used to evaluate the cost-effectiveness of different survey methods, they do not provide sufficient data to generalize the cost of conducting these surveys at the regional or global level. In this paper, we present an analysis of costs incurred in the implementation of trachoma prevalence surveys across eight national trachoma control programs. The findings from this analysis will enable national trachoma program managers and international partners to budget for trachoma prevalence mapping appropriately.

## Methods

### Ethics Statement

The analysis of prevalence survey cost data did not involve any research on human subjects. The prevalence surveys reviewed in this paper were conducted in accordance with the Declaration of Helsinki and reviewed by the Emory University Institutional Review Board or the London School of Hygiene and Tropical Medicine (LSHTM) Ethical Committee and each country's respective Ministry of Health. External funding for the prevalence surveys was as follows: LSHTM, The Gambia survey; Helen Keller International, Sikasso Region of Mali; The International Trachoma Initiative and The Carter Center, 18 districts in Ghana; The Carter Center, all other surveys.

### Data Collection

A systematic review of trachoma prevalence surveys conducted in Ethiopia, Ghana, The Gambia, Mali, Niger, Nigeria, Sudan, and Southern Sudan was performed February through May 2010. This review of prevalence survey costs included surveys that employed a PBPS methodology to estimate trachoma prevalence at the district level, or the administrative unit equivalent to a district (administrative unit with population of approximately 100–250 thousand people: *woreda* in Ethiopia, region in The Gambia, local government area in Nigeria, locality in Sudan, and county in Southern Sudan). Included surveys were implemented from 2006–2010, and funded or co-funded by The Carter Center, LSHTM (The Gambia), The International Trachoma Initiative (Ghana), or Helen Keller International (Sikasso Region, Mali). All surveys were ‘cluster random surveys’ that used a two stage sampling process to select clusters (communities, villages, or enumeration areas) representative of the domain in the first stage and households within the cluster in the second. The numbers of clusters and households in the surveys was not constant between districts.

A data collection tool was used to collect the actual costs incurred in local currency during survey activities from accounting records in the programs. The tool collected data for four cost activities: training, field work, supervision and data entry. Training included costs such as *per diem* of trainees and trainers, meeting facility and supplies, transportation to the practical exercise and any required overnight accommodation. Field work costs included *per diems* for survey personnel (trachoma grader and recorder), transportation of survey field team, accommodation and supplies such as tetracycline eye ointment and magnifying loupes. Supervision included any *per diem*, transport and accommodation paid to Ministry of Health or NGO personnel retained for supervision of field work activities. Data entry costs included *per diem* of data entry clerks, cost of computer rental and information technology support (if required) and supplies. For each cost activity, data were collected on the number of people paid, the daily rate and the number of days paid. Transportation costs included any vehicle rental, fuel expense and driver *per diem*.

The data collected in this study captured the incremental cost of conducting prevalence surveys in the context of an existing national trachoma control program. Ministry of Health and NGO salaries and other associated costs were not included in the analysis. Integrated prevalence surveys (more than one disease measured) were excluded from this analysis. “Headquarters” expenses were not included in the primary analysis of prevalence survey costs. Although beneficial, consultant or other outside technical assistance is not required for a national program to conduct trachoma prevalence surveys. Furthermore, the cost of outside technical assistance is dependent on travel expense policies which are unique to each partner. The cost of Carter Center headquarter support for specific survey activities are reported in this review, but were not included in the district-level cost data, as these costs are organization-specific and cannot be generalized.

Once completed, the cost data forms were verified against the financial reports from the Carter Center, Helen Keller International, LSHTM or the Ministries of Health. In Ghana, Ethiopia and Northern Sudan, exact data on distance traveled were not available; the data reported for these programs' distance traveled are estimates from the national programs.

### Data Analysis

Data were converted to US dollars using the mean of the weighted average exchange rate from the World Bank (http://data/worldbank.org/indicator/PA.NUS.FCRF) for the years 2007–2009. Since most district-level prevalence surveys were conducted in groups (i.e. all districts in a region surveyed at the same time), costs were not reported for each individual district. Rather, each “grouping” of surveys that were financed at the same time was analyzed as the same observation. For example, in the Kayes Region of Mali, all 7 districts were surveyed using the same survey personnel within the same period of time. Funds were provided to the Ministry of Health to conduct the survey work for the entire region, which resulted in efficiencies gained by conducting one initial training and reducing the amount of transport required. Where data were reported in this fashion, the districts are treated as the same observation in the analysis.

Based on these observations, the analysis generates the overall costs, the average survey costs per district and average costs per cluster for each observation. Data were first entered into Excel and then analyzed using STATA to generate descriptive statistics for each cost activity. Subsequently, a cost composition analysis was performed. The data were classified into activities as defined in the data collection tool to calculate the proportion of the total cost for each cost activity. Within each of the four activities (training, field work, supervision and data entry), four main cost categories were identified: personnel, transportation, supplies and other. The costs for each category were compared against the total cost for each activity to identify the main cost drivers of survey expenses.

Normally distributed data are presented as the mean and standard deviation (SD). Not-normally distributed data is presented by the median and inter-quartile range (IQR).

## Results

### Survey Costs

**Figure 1:**
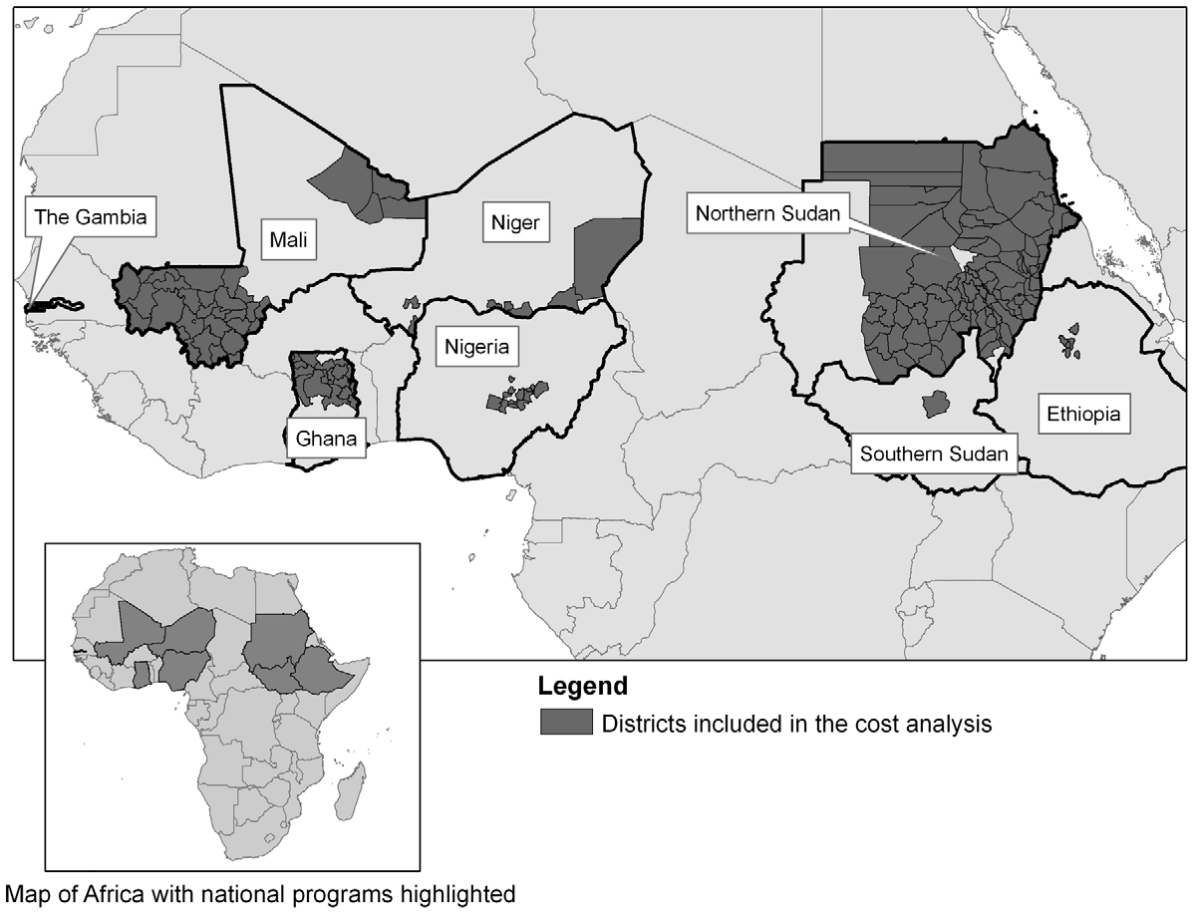
Map of district-level trachoma prevalence surveys included in the cost analysis.

A total of 29 observations were collected from eight national trachoma control programs. The cost per district by observation is presented in Table 1. Overall, a total of 165 district-level surveys were included (Figure 1), representing a total of 3,203 clusters surveyed. The average costs per district were skewed to the right by an outlier (Ayod in Southern Sudan, $25,409) so are described by the median, $4,784 and IQR, $3,508–$6,650. The median cost per cluster was $311 (IQR = $119–$393) whilst the median cost per person screened was $3.50 (IQR = 1.94–4.16). (The mean cost per district, cluster and person was $5,849 (SD = $4,635), $324 (SD = $236), and $3.39 (SD = $2.02) respectively). The least expensive survey per district was in Ethiopia, approximately $1,511 per district. The number of districts, clusters and persons sampled per observation is presented in Table 1.

**Table 1:** Summary of total costs, by observation.

National program	Observation	Number of districts	Number of clusters	Number of households per cluster	Number of people examined	Total costs ($)	Cost per district ($)	Cost per cluster ($)	Cost per person screened ($)	Reference
Ghana	Northern & Upper West	18	720	30	74,225	72,249	4,014	100	0.97	Yayemain 2009
Mali	Kidal	1	20	24	2,165	14,777	14,777	739	6.83	Bamani 2010
	Kayes	7	140	24	13,576	13,593	1,942	97	1.00	Bamani 2010
	Koulikoro	9	180	24	19,342	17,505	1,945	97	0.91	Bamani 2010
	Sikasso	8	160	24	18,795	19,046	2,381	119	1.01	PNLCC
	Segou	8	160	24	16,471	18,553	2,319	116	1.13	PNLCC
Nigeria	Plateau & Nasarawa	13	260	16	21,606	24,036	1,849	92	1.11	King 2010
Southern Sudan	Jonglei (Ayod County)	1	20	20	2,335	25,409	25,409	1,270	10.88	King 2008
Northern Sudan	Kassala	10	132	30	10,576	35,308	3,531	267	3.34	FMOH GOS
	Blue Nile	4	45	20	5,166	18,799	4,700	418	3.64	FMOH GOS
	Gazeira	7	105	20	10,466	42,049	6,007	400	4.02	FMOH GOS
	White Nile	8	120	20	10,570	39,168	4,896	326	3.71	FMOH GOS
	Gadarif	10	150	20	13,682	47,839	4,784	319	3.50	FMOH GOS
	Sinnar	7	105	20	9,095	34,961	4,994	333	3.84	FMOH GOS
	River Nile	6	90	20	7,528	20,632	3,439	229	2.74	FMOH GOS
	Red Sea	10	150	20	9,918	40,680	4,068	271	4.10	FMOH GOS
	Northern	5	66	20	11,076	36,454	7,291	552	3.29	FMOH GOS
	North Kordofan	9	135	20	10,360	37,494	4,166	278	3.62	FMOH GOS
	South Kordofan	9	135	20	10,755	41,960	4,662	311	3.90	FMOH GOS
Niger	Magaria	1	20	24	1,789	7,884	7,884	394	4.41	PNLCC Niger
	Matameye	1	20	24	1,712	7,835	7,835	392	4.58	PNLCC Niger
	Nguigmi	1	20	24	1,659	7,866	7,866	393	4.74	PNLCC Niger
	Maine Soroa	1	20	24	1,867	7,866	7,866	393	4.21	PNLCC Niger
	Maradi Commune	1	20	24	2,393	6,132	6,132	307	2.56	PNLCC Niger
	Tessaoua	1	20	24	1,806	6,132	6,132	307	3.40	PNLCC Niger
	Gaya	1	20	24	2,036	6,650	6,650	333	3.27	PNLCC Niger
	Loga	1	20	24	1,801	6,650	6,650	333	3.69	PNLCC Niger
Ethiopia	Amhara	5	90	10	5,762	7,556	1,511	84	1.31	Ngondi 2008
The Gambia	Lower River & North Bank	2	60	25	2,990	7,815	3,908	130	2.61	Harding-Esch 2009
Total		165	3,203		301,552	672,897				

### Composition of Survey Costs

When the costs for each survey activity were compared against the total cost (Table 2), the data showed that field work comprised on average 69.9% of the total cost of a survey. Among the observations, the proportion of total costs spent on field work ranged from 44.9% to 90.5%. Training costs ranged from 1.0% to 29.6% of total costs, supervision expenses were between 0.0% and 20.9% of the total, and data entry costs ranged from 0.0% to 25.0% across all observations. Within each survey activity, personnel costs were the most expensive, with personnel costs in field work accounting for 40.4% of the total survey costs reported by the national programs, followed by transportation during field work at 22.4%.

**Table 2:** Average proportion of total survey costs attributed to cost categories and activities.

	Activities				
	Training	Field work	Supervision	Data entry	Total
Category					
Personnel	1.9%	40.4%	11.3%	10.9%	64.6%
Transportation	1.6%	22.4%	1.7%	0.0%	25.7%
Supplies	0.9%	5.3%	0.0%	0.0%	6.3%
Others	1.4%	1.7%	0.3%	0.0%	3.3%
Total	5.9%	69.9%	13.2%	10.9%	100.0%

Training and data entry activity costs were reported by observation as the cost for each activity. These costs were not always directly related to the number of districts surveyed as some programs did not incur cash costs for these activities. The mean cost of training was $1,342 (SD $659) while the median was $1,791.50 (IQR = $588–$1,816). The mean cost of data entry was $2,548 (SD $3,493) and the median was $1,028 (IQR = $415–$4,431).

### Costs of ‘Headquarters’ Participation in Surveys

Although the cost of outside technical assistance was not factored into the district or cluster level cost analysis, there were 9 observations that were surveyed with at least one representative from The Carter Center Headquarters (Atlanta, Georgia, USA) present, covering a total of 58 districts. The average cost for airfare, hotel, meals and incidentals per person-trip was $1,779 (n = 13, SD = $2,027) from 2006–2010.

## Discussion

It is possible that trachoma control programs do not implement prevalence surveys due to a perception that the costs will be beyond the capacity of the program. However, the results of this analysis show that such surveys are not cost-prohibitive. The range of costs per district varied from $1,151–$25,409, in large part due to differences in accessibility and the number of clusters sampled in each survey. Of the 29 observations, only three surveys reported a cost per cluster exceeding $500: Ayod in Southern Sudan, Kidal in Mali and the Northern Region in Sudan. These surveys were characterized by both high transport and personnel costs. In Ayod County of Southern Sudan, where the average cost per cluster was $1,270 and average cost per person screened was $10.88, vast distances of water-logged and unforgiving terrain made vehicle transport impossible, requiring a chartered airplane to transport staff to airstrips from where they traveled to the clusters on foot over a period of days. These exceptional circumstances therefore required additional staff, working for a longer period of time, and transport by chartered aircraft. In Kidal Region (a desert region of Mali), the second most expensive survey per cluster ($739 per cluster, $6.83 per person screened), the sparse population (80,000) and low population density (less than one person per square kilometer) resulted in the national program treating the region as the domain, with the consequence that the distances between clusters was hundreds of kilometers. To conduct this survey, the program rented vehicles instead of using Ministry of Health and NGO transport due to security concerns in the area. The Northern Region of Sudan ($552 per cluster, $3.29 per person screened) is also on the edge of the Sahara with similar demands on transport and time. Least expensive, at under $100 per cluster, were the surveys conducted in the Amhara region of Ethiopia ($84 per cluster, $1.31 per person screened) and Plateau and Nasarawa States of Nigeria ($92 per cluster, $1.11 per person screened) where *per diem* rates were low and the population is relatively dense, reducing both the travel costs and time spent travelling between clusters. In total, 7 observations cost less than $125 per cluster and these also had the lowest cost per person screened ($0.91–$1.31). In these surveys, the relative proximity of clusters and low *per diem* rates contributed to lower costs in comparison to the more expensive surveys.

Among the cost categories reported, the *per diem* of field staff and supervisors and the cost of transportation accounted for 73% of the total survey costs. In settings where distances between communities are great, trachoma control programs may consider reducing the number of clusters surveyed and increase the number of people screened per cluster to reduce costs but maintain an adequate sample size. However, the risks to accuracy and precision around the prevalence estimate should be considered. Cost savings on transport and accommodation costs can be achieved by planning the route of vehicles between clusters carefully. A route for two teams can often be planned in which the teams share one vehicle, work in the first and second clusters simultaneously (with the vehicle shuttling between as necessary) and then travel together to the next cluster where they camp for the night and sensitize the village population of the survey to be conducted the following day. Such transport sharing and camping has been both effective and enjoyable in most of the countries in this analysis. *Per diem* and allowance costs vary by national program, level of trained personnel recruited to serve as survey team members and local supervision requirements. *Per diem* costs in the surveys studied ranged from $6.21 per day for graders (junior health staff) to $250 a day for senior supervisors (an ophthalmology professor and National Coordinator). When designing surveys, due consideration should be given to assign roles and responsibilities consistent with the qualification and *per diem* given. Junior health staff who are comfortable with the climate, social circumstances and geography of the area to be surveyed make ideal field staff, and serve to lower *per diem* costs. It is appropriate for a National Coordinator or ophthalmology professor to spend a day or two testing the ability of the trained examiners before the survey starts, but costs can be reduced if that person does not spend many days in the field.

The review of data entry costs also presents new findings for Ministries of Health. Although data entry was not an expense for all surveys reported, data entry accounted for an average of 11% of total survey expenses. In this sample, the incremental cost of data entry ranges from 0% in surveys where existing program staff conducted data entry on existing computers incurring no additional cash cost to 25% of the total cost of the survey where external contractors were hired to complete the work. Survey planners should consider the cost of data entry in their own country context to ensure that costs for double entry, analysis and preparation of printed reports are included in budgets.

By design, we did not capture the cost of each Ministry of Health and NGO employee who contributed time to conduct survey work, the incremental cost effectiveness ratio is likely to be underestimated since these costs were not taken into account. This could be included in the analysis as an opportunity cost. However, since the implementation of prevalence surveys is recommended as the standard monitoring and evaluation framework for trachoma control programs by the WHO, these surveys were within the mandate of the Ministry of Health personnel who were engaged in field work and supervision. Salary costs were excluded as they were considered part of the functional trachoma control program and we sought to establish the incremental cost of conducting surveys in the presence of a program. We also did not include the cost of technical assistance (including travel) for ‘headquarters’ staff. Although the average cost of a person-trip from The Carter Center for technical assistance was $1,779 (SD = $2,027), we considered this to be a non-essential cost for a program, subject to considerable variation between supporting NGOs who have different travel policies, and likely to come from a different operating budget which would not have an incremental effect on the cost of a national program.

The selection of a sample representative of the underlying population presents an opportunity to collect data on multiple conditions and this has been done for trachoma and malaria[11] and trachoma and urinary schistosomiasis[12]. Such integrated surveys were not included in this analysis since they were considered special cases and not what is typically done. However, the costs of adding indicators for additional diseases or conditions are the additional personnel, equipment and consumables required for that survey, with the other cost items such as transport and *per diem* of the drivers and assistants covered by the ‘parent’ survey.

Although the data presented show costs from a variety of settings, there are a few limitations. The data in this analysis were reported retrospectively and therefore, it is possible that some costs may not have been captured. For some surveys (Ghana, Ethiopia and Northern Sudan) log book entries for distance travelled were not available and we relied on the local knowledge of the national program to calculate distance travelled. Each of these surveys was conducted in the presence of a functioning trachoma control program; there was no need to purchase new vehicles or make other large capital expenses. Survey work performed in the absence of this infrastructure would be more expensive. New country programs may find it necessary to rent vehicles and seek technical assistance for training survey staff, the costs of which would need to be considered in addition to the incremental costs of conducting a survey presented here.

There are variations in the number of clusters surveyed among the different observations, based on the population of each survey domain, which may affect the comparability of the survey costs among different countries. However, the authors expected variation among national programs due to differences such as *per diem* rates, the level of qualified health professional involved in field work, and the capacity to complete data entry. The variation seen in these data illustrate the context-specific nature of planning survey activities. However, these limitations should not discourage program managers from using the data presented in this paper as benchmarks for determining funding needs.

Twenty-six out of the 29 observations were conducted with external funding exclusively from The Carter Center, which may imply the cost estimates are limited to those surveys supported by this NGO. However, there are similarities between the cost per cluster from The Gambia, which was fully funded by LSHTM, districts in Mali supported by Helen Keller International, and districts in Ghana co-sponsored by the International Trachoma Initiative and The Carter Center. This suggests that our findings are not unique to the operating principles of one NGO.

Since transport and *per diem* were identified as major cost drivers, it is possible to predict total survey costs for areas requiring surveys. It is also possible to use these data to project the cost of other survey methodologies by applying the average cost per cluster to the number of clusters required. Despite the potential limitations of this study, these data present the only summary of actual costs incurred during trachoma prevalence surveys in the peer-reviewed literature. For the goal of elimination of blinding trachoma worldwide by 2020 to be met, national programs will need to budget for impact evaluation at the district level. The cost of epidemiologically rigorous surveys should not been seen as a barrier to their implementation. With adequate baseline and impact evaluation data, national programs can maximize their limited programmatic resources. These data should inspire national trachoma program managers and ministry of health staff involved in other public health supervisory roles to consider implementation approaches that ensure surveys are designed in a cost-effective and efficient manner. These cost data will enable the international trachoma control community to create global estimates on the cost to complete trachoma prevalence mapping and estimate the financial needs to support impact assessments to measure progress towards the elimination of blinding trachoma.
